# Post-Myocardial Infarction (MI) Left Ventricular Free Wall Rupture Managed Conservatively

**DOI:** 10.7759/cureus.64395

**Published:** 2024-07-12

**Authors:** Nikhale Malik, Abhishek Vadher, Kurian Panikottu, Sujata Kambhatla, William Harder

**Affiliations:** 1 Internal Medicine, Garden City Hospital, Garden City, USA; 2 Cardiology, Garden City Hospital, Garden City, USA

**Keywords:** oozing left ventricular free wall rupture, conservative treatment, complication of myocardial infarction, left ventricle free wall rupture, st-elevation myocardial infarction (stemi)

## Abstract

Left ventricular free wall rupture (LVFWR) is an uncommon but often fatal complication of acute myocardial infarction. LVFWR is managed with hemodynamic stabilization and is typically followed by surgical intervention with varying approaches depending on the type of LVFWR. A 78-year-old male with a history of coronary artery bypass graft (CABG) was admitted with ST-segment elevation myocardial infarction. Left heart catheterization showed complete occlusion of the saphenous vein graft to the 1st obtuse marginal artery. The patient was not a candidate for percutaneous coronary intervention or CABG. The patient later developed atrial fibrillation with a rapid ventricular response which was managed with beta blockers. Computed tomography pulmonary angiogram was done to rule out pulmonary embolus; however, it demonstrated findings of a lateral LVFWR. The patient was deemed a poor surgical candidate for cardiothoracic surgery, and the LVFWR was managed conservatively with metoprolol succinate and bed rest. He later required amiodarone and direct current cardioversion due to the recurrence of atrial fibrillation. Two months following the LVFWR, the patient remained stable with no apparent complications. In a certain subset of LVFWR patients, surgical management may not be possible given patient anatomy and other high-risk factors. In these cases, conservative management with bed rest and beta blockers and treatment of ventricular and atrial arrhythmias may be a viable therapeutic option.

## Introduction

Mechanical complications of acute myocardial infarction (AMI), such as left ventricular free wall rupture (LVFWR), ventricular septal perforation, and mitral valve chordae tendineae rupture, are uncommon but major contributors to post-myocardial infarction (MI) mortality. The incidence of LVFWR following AMI ranges from 0.8% to 6.2% and has decreased significantly with widespread adoption of percutaneous coronary intervention (PCI) [1]. Risk factors for the development of LVFWR following AMI include female gender, age>60, no history of angina, hypertension without left ventricular hypertrophy, and anterior location of the AMI [2-3]. Typically, LVFWR occurs within 2-4 days following AMI [4]. LVFWR remains one of the most fatal complications of AMI with a fatality rate estimated between 75% and 90% [5]. 

LVFWR presents with signs and symptoms that may include dyspnea on exertion or at rest, syncope, persistent chest pain, nausea, vomiting, and cardiogenic shock and can potentially progress to electromechanical dissociation leading to sudden cardiac death. Typically, LVFWR requires intervention which may include linear closure, infarctectomy and closure with bioprosthetic patch, or percutaneous intra-pericardial fibrin glue injection [6]. Given its high mortality rate, it is exceedingly rare for LVFWR to be managed conservatively. In this case report, we describe an elderly male who developed LVFWR post-AMI and PCI who was managed conservatively without complications or adverse outcomes. 

## Case presentation

**Figure 1:**
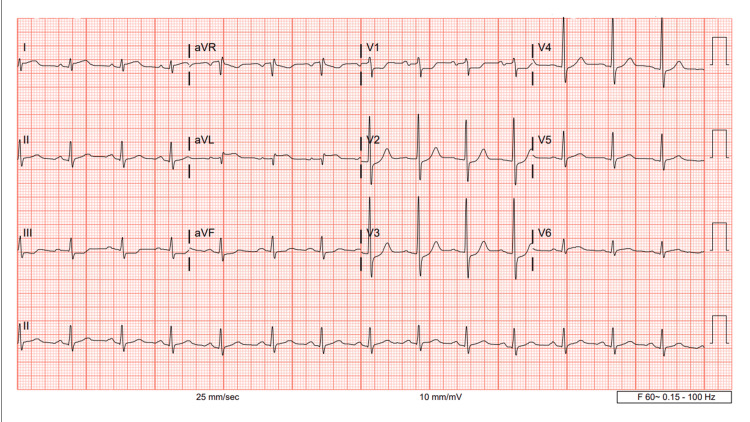
Electrocardiogram demonstrating ST elevations in aVL and ST depressions in leads III, aVF, and V1-V3.

A 78-year-old male with a past medical history of coronary artery disease, hypertension, hyperlipidemia, coronary artery bypass graft (CABG) done 12 years ago, percutaneous coronary intervention with two drug-eluting stents deployed to saphenous vein graft (SVG) to the right posterior descending artery (RPDA), presents to the hospital with a complaint of sharp and crushing substernal chest pain which started when he was showering. Initial Troponins were 5490 ng/L and then increased to 7536 ng/L in three hours. On admission, an electrocardiogram (EKG) showed ST elevations in leads I and aVL (Figure 1). The cath lab was activated, and the patient was taken for emergent left heart catheterization (LHC). 

**Figure 2:**
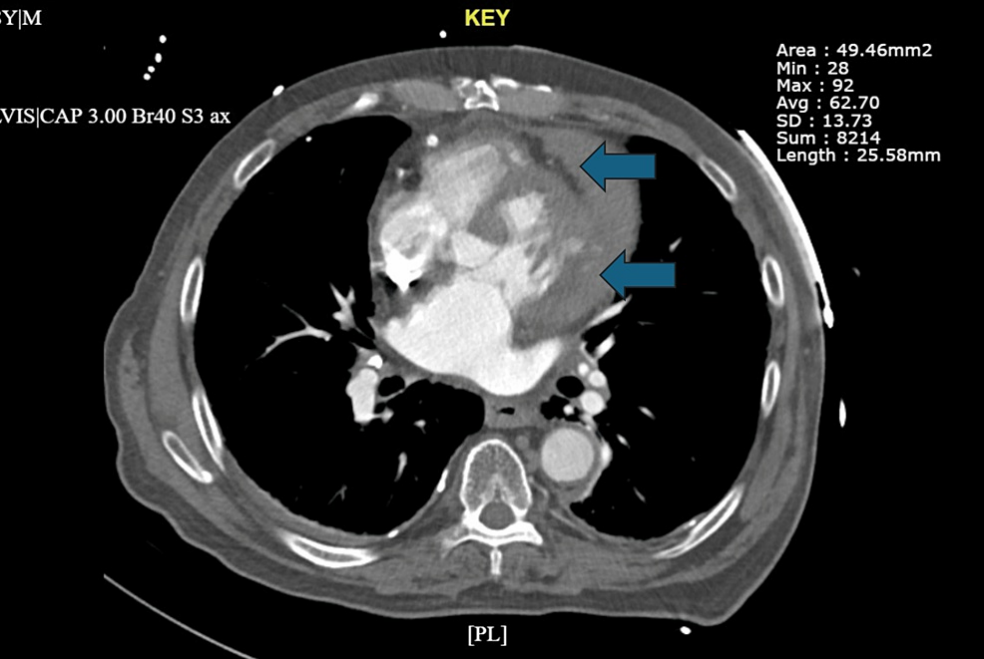
Computerized tomography pulmonary angiography was performed demonstrating a moderate pericardial effusion (indicated by the blue arrows) arising from the left ventricle. The density of the pericardial effusion is 61.85 Hounsfield units, indicative of hemorrhage.

LHC was performed and showed complete occlusion of the SVG to the 1st obtuse marginal artery (OM1) beginning in the ostial segment. SVG to RPDA graft was patent with 70% in-stent stenosis of proximal SVG stent with thrombolysis in MI flow 3. The initial plan was to transfer to a tertiary care center for a CABG but after discussion with the cardiothoracic surgery team, the patient was deemed not a candidate for CABG given the absence of graftable targets. An intra-aortic balloon pump (IABP) was placed to augment the blood flow through the coronaries. Heparin infusion was continued along with aspirin, ticagrelor, and atorvastatin. The patient’s chest pain was resolved with medical therapy. Two days after the LHC, the IABP was removed, and the heparin infusion was stopped after a total duration of 48 hours. On the third day of admission, the patient developed acute dyspnea and chest pain, became hypotensive and tachycardic with a new onset of atrial fibrillation with a rapid ventricular response (RVR), and had a low-grade fever. A CT-pulmonary angiogram was performed demonstrating no evidence of pulmonary embolism, but a defect was noted in the lateral LV free wall with contrast extravasation into the pericardial space (Figure 2). Imaging confirmed the diagnosis as LVFWR arising from the lateral LV wall in the territory of the patient’s acute SVG-to-OM1 occlusion. 

The patient was immediately transferred to a tertiary care center for surgical evaluation. Hemodynamics stabilized and his symptoms resolved. He was managed conservatively overnight and on the next day, a transthoracic echocardiogram was repeated, showing a fibrinous pericardial effusion without cardiac tamponade which likely represents clotted blood. Cardiothoracic surgery was consulted, and they recommended non-operative management due to his advanced age, recent MI, and high intraoperative risk. A transesophageal echocardiogram was performed and showed moderate pericardial effusion containing a fibrinous material which is consistent with blood. The effusion appeared to originate from a contained free wall rupture in the inferolateral wall with no remaining flow visualized either with color flow and definite contrast between LV and the pericardial space. As there was no active extravasation into the pericardial space, this indirectly indicates that a thrombus has formed. Later in his hospital stay, he re-developed atrial flutter with the RVR requiring direct current cardioversion. On discharge, the plan was made to start oral apixaban two weeks post-MI presentation, for a duration of three weeks, and then transition to oral aspirin after a thorough discussion of the risks and benefits of the treatment options with the patient given the risk of stroke after cardioversion. The patient was at high risk for ventricular aneurysm development and re-rupture and hence a poor candidate for long-term oral anti-coagulation. Two months following the LVFWR, the patient remained stable with no apparent complications. 

## Discussion

Though rare, LVFWRs remain one of the most fatal complications of AMI. Appropriate management of LVFWRs can vary based on the type of LVFWR, location, patient demographics, presence of collateral blood flow, and several other factors. Generally, patients with LVFWRs require inotropic support, IV fluids, and IABP. The IABP can reduce intraventricular pressure thereby decreasing the risk of further infarction and expansion of the LV defect [7]. Pericardiocentesis may be indicated in cases where the LVFWR is complicated by cardiac tamponade; however, it is not routinely performed as it can impede clot formation over the area of LV rupture. 

LVFWRs can be broken down into two major categories: “blowout” and “oozing”. The blowout subtype is defined as having a macroscopic defect in the epicardium with free communication between the LV and pericardium, and the oozing subtype is defined as a microscopic defect characterized by epicardial extravasation or slow bleeding into the pericardium. Blowout LVFWRs are associated with a higher intraoperative and overall mortality rate and a significantly higher incidence of complications including true ventricular aneurysms in comparison to the oozing subtype [8].

Though historically oozing-type LVFWRs have been treated using sutured techniques, they are increasingly being treated with sutureless repairs which can include using collagen patches, xenopericardium (commonly bovine), and fibrin glue [9-11]. While collagen patches may have excellent adaptability, given the nature of a sutureless approach there is an inherent risk of repeat LVFWR. One meta-analysis demonstrated a 22.4% risk of 30-day mortality in sutureless techniques and a 38.4% risk in sutured [7]. 

Following surgical intervention, the risk of repeat LVFWR is high. With our patient, it was decided by the team of cardiothoracic surgeons that the patient is at a very high risk for hemodynamic instability secondary to the frail myocardial tissue due to a recent MI a week prior to admission, his advanced age, and his prior cardiac history. With regard to the patient’s prior sternotomy, it may have been protective as prior sternotomy is associated with pericardial adhesions which likely acted to contain his LVFWR and prevent tamponade. At the same time, pericardial adhesions can increase the surgical difficulty of access to the heart and thus increase the risk of complications during surgical manipulation. Hence, it was decided to avoid the surgery and manage him medically. 

The location of the wall rupture plays a major role in deciding whether the patient is a candidate for conservative medical management or surgical intervention. It is believed that in a posterior wall lesion, the weight of the heart contributes to hemostasis [12]. A prior study demonstrated that medical management might be a good choice in patients with lateral and inferoposterior AMI or high-risk surgical candidates like patients with age >75 years, or large infarct areas [13]. Even though the patients are being managed conservatively, such patients should be closely followed by a cardiothoracic surgeon since these patients are always at a very high risk of re-rupture or metachronous double rupture [14]. 

It is believed that the reduction in myocardial contractility and lowering of blood pressure with beta blockers appear to reduce the incidence of re-rupture of the free wall [15]. Furthermore, an emphasis should be placed on minimizing physical activity and encouraging bed rest as this also reduces strain on the heart and reduces the chances of re-rupture [15]. In many cases, uncontrolled hypertension can contribute to re-rupture, and the use of beta blockers can assist with blood pressure under control [15]. Once out of the acute phase of the LVFWR and discharged home, these patients still remain at substantial risk of ventricular aneurysms [15]. 

## Conclusions

In conclusion, certain LVFWR patients may not qualify for surgical intervention given a multitude of comorbidities which may include advanced age and recent thoracic surgery. As with our patient, beta blockers, bed rest, close patient monitoring, treatment of complications, and serial echocardiograms may be viable therapeutic options in a certain subset of patients. With this case report, we present a rare case of LVFWR which was managed conservatively with no complications two months following the rupture.
